# ^99m^Tc-MIBI uptake as a marker of mitochondrial membrane potential in cancer cells and effects of MDR1 and verapamil

**DOI:** 10.1371/journal.pone.0228848

**Published:** 2020-02-12

**Authors:** Jin Won Park, Sun-pyo Hong, Jin Hee Lee, Seung Hwan Moon, Young Seok Cho, Kyung-Ho Jung, Jeeyun Lee, Kyung-Han Lee

**Affiliations:** 1 Department of Nuclear Medicine, Samsung Medical Center, Sungkyunkwan University School of Medicine, Seoul, Korea; 2 Department of Health Science and Technology, Samsung Advanced Institute for Health Sciences & Technology, Sungkyunkwan University, Seoul, Korea; 3 Department of Medicine, Samsung Medical Center, Sungkyunkwan University School of Medicine, Seoul, Korea; Columbia University, UNITED STATES

## Abstract

We investigated the relation of ^99m^Tc-MIBI uptake to mitochondrial membrane potential (MMP) in cancer cell lines and patient-derived tumor cells (PDCs). In T47D and HT29 cells with low MDR1 expression, FCCP dose-dependently reduced MMP and ^99m^Tc-MIBI accumulation in similar patterns with nearly perfect linear relationships. T47D and HT29 cells with high MDR1 expression had low ^99m^Tc-MIBI accumulation that was minimally affected by FCCP dose. In these cells, verapamil markedly increased ^99m^Tc-MIBI accumulation to magnitudes that were excessive compared to MMP increase. Decreased plasma membrane potential by verapamil and its recovery by FCCP suggested that enhanced ^99m^Tc-MIBI transport through modified plasma membranes contributed to the excess accumulation. Evaluation of three different colon cancer PDCs with low to modest MDR1 expression verified that FCCP significantly suppressed MMP and similarly reduced ^99m^Tc-MIBI accumulation. Verapamil partially recovered both MMP and ^99m^Tc-MIBI accumulation that was lowered by FCCP. Importantly, a high linear correlation was found (r = 0.865) between ^99m^Tc-MIBI accumulation and MMP in these cells. These findings indicate that low baseline ^99m^Tc-MIBI uptake that is markedly increased by verapamil represents cancer cells with high levels of MDR1 expression. However, in cancer cells with low or modest levels of MDR1 expression that do not markedly increase ^99m^Tc-MIBI uptake by verapamil, the magnitude of uptake is largely dependent on cellular MMP.

## Introduction

Tumor imaging with the lipophilic cation ^99m^Tc-sestamibi (^99m^Tc-MIBI) is considered a noninvasive method for detecting multidrug resistance [1–3]. ^99m^Tc-MIBI is recognized as a substrate for P-glycoprotein, an ATP-dependent transmembrane transporter encoded by the multidrug resistance 1 (MDR1) gene. This transporter actively pumps out specific substrates including chemotherapeutic agents, conferring cancer cells resistance to a wide range of anticancer drugs [4]. Despite the focus on the potential ability of ^99m^Tc-MIBI imaging to assess tumor MDR1 expression, its appropriate use requires the recognition that tumor accumulation of the tracer is influenced by other biological factors. Indeed, some studies have failed to show that tumor ^99m^Tc-MIBI uptake is correlated to MDR1 expression [5], while others found that it was not useful for predicting chemotherapy response [6]. Whereas MDR1 promotes the washout of ^99m^Tc-MIBI after it is taken up, without sufficient initial uptake, there would be little ^99m^Tc-MIBI in the cells to begin with. Therefore, low tumor ^99m^Tc-MIBI activity does not necessarily indicate MDR1-mediated efflux but may also represent poor tracer uptake [7].

The key driver for the uptake of cell-permeant cationic probes is mitochondrial membrane potential (MMP) [8–12]. Mitochondria generate an electrical gradient by delivering protons via redox carriers to the inner membranous space, which results in a negative membrane potential. The magnitude of this mitochondrial electrical gradient is the force that drives cellular uptake of cationic ^99m^Tc-MIBI [10–11]. Cancer cells are associated with a significantly greater MMP [13–17] that contributes to tumor progression [13]. Higher MMP of cancer cells may also contribute to resistance against apoptotic cell death [14–17], in which dissipation of the mitochondrial electrical gradient is a critical event [14]. It would therefore be beneficial to clarify how cancer cell MMP influences tumor ^99m^Tc-MIBI uptake.

The effect of MMP on cancer cell ^99m^Tc-MIBI accumulation is confounded in the presence of extreme levels of MDR1 protein, wherein measurements need to be evaluated while blocking tracer efflux. The most widely used P-glycoprotein modulator for this purpose is verapamil. Indeed, the standard criterion of positive MDR1 function using ^99m^Tc-MIBI is increased accumulation in the presence compared to absence of verapamil [18–22]. In this study, we thus investigated the relationship between ^99m^Tc-MIBI uptake and MMP in cancer cells with low and high levels of MDR1 expression, in the presence and absence of verapamil. MMP level was modulated using carbonyl cyanide-p-trifluoromethoxyphenylhydrazone (FCCP), a representative protonophore that facilitates proton transport across the mitochondrial membrane to short-circuit the electrochemical gradient [23]. Four different colon cancer cell lines and three different colon cancer patient-derived tumor cells (PDCs) were evaluated in this study.

## Materials and methods

### Materials and cell culture

MIBI (Cardiolite^TM^) was from Mallinckrodt Pharmaceuticals (UK). Verapamil (P-glycoprotein inhibitor), MK-571 (MRP1/2 inhibitor), FCCP and tetramethylrhodamine were from Sigma (St. Louis, MO). Mitotracker Red FM was from Invitrogen^TM^ (Carlsbad, CA), and DiBAC4(3) was from Molecular Probes^®^ (Eugene, OR). Polyclonal antibodies against MDR1 and MRP1 were from Abcam (Cambridge, MA). EFLUXX-ID Green multidrug resistance assay dye was from Enzo Life Sciences, Inc. (Lörrach, Germany).

T47D human breast cancer cells, HCT15 and HT29 human colon cancer cells, and CT26 mouse colon cancer cells were from the American Type Cell Culture. Cells were maintained in RPMI 1640 media (Lonza; Swiss) supplemented with 10% fetal bovine serum (Serena; Germany), 2 mM L-glutamine, and penicillin-streptomycin at 100 U/ml in 5% CO_2_ at 37°C. Cells of 70–80% confluence were used for experiments. PDCs were obtained from malignant ascites, pleural or pericardial effusions collected from patients with metastatic colon cancer as previously described [24]. Briefly, the collected effusion fluid was centrifuged at 1500 rpm for 10 min and washed twice with phosphate buffered saline (PBS). Cell pellets were resuspended in culture medium and plated into culture flasks. All procedures were carried out according to guidelines from the Declaration of Helsinki. The patients gave written informed consents, and the Institutional Review Board approved the protocol for this study.

### Sulforhodamine-B (SRB) assays for measurement of viable cell content

Cells were fixed with 10% (w/v) trichloroacetic acid and stained with SRB for 30 min. Excess dye was removed by repeated washing with 1% (v/v) acetic acid, and protein-bound dye was dissolved in 10 mM Tris base solution for determination of optical density at 510 nm on a micro-plate reader.

### Immunoblotting for MDR1 and MRP1 expression

Cells were washed with PBS and solubilized in PRO-PREP^TM^ protein extraction solution (iNtRON biotechnology; Korea) for 15 min at 4°C, and cell debris was eliminated by centrifugation at 14,000 rpm for 10 min at 4°C. The supernatant was analyzed for protein content by the Bradford method, and 40–60 μg of protein were separated on a 10% polyacrylamide gel. Protein was transferred to a PVDF membrane (Millipore; Burlington, MA) and incubated overnight at 4°C with polyclonal antibodies against MDR1 (1:1000) or MRP1 (1:250) in tris-buffered saline (50 mM Tris, pH 7.5, 150 mM NaCl) containing 0.05% Tween 20 and 5% skim milk. After washing 3 times for 10 min with tris-buffered saline containing Tween 20, the membrane was incubated with secondary antibodies (1:2000) for 1 h at room temperature. Immune reactive protein was finally detected with an enhanced chemiluminescence kit (Thermo Fisher Scientific; MA).

### Measurement of ^99m^Tc-MIBI accumulation

MIBI was labelled with ^99m^Tc according to the manufacturer's instructions. Cells were plated on 24-well flat-bottomed plates in 0.5 ml of medium and allowed to grow for 2 days. Cells were pre-incubated for 20 min at 37°C in culture media with FCCP or verapamil. After incubation with 185 kBq of ^99m^Tc-MIBI in 0.5 ml of medium for 20 min in 5% CO_2_ at 37°C, cells were rinsed twice in ice-cold PBS and then lysed with 0.1 N NaOH. Cell-associated radioactivity was recovered and measured using a high energy gamma counter (Perkin Elmer; MA), which was expressed as % uptake per well relative to controls.

### Measurement of P-glycoprotein activity

Measurement of P-glycoprotein activity using Efluxx-ID Green multidrug resistance assay dye was performed as previously reported [25]. This hydrophobic compound readily penetrates the cell membrane and is hydrolyzed to a hydrophilic fluorescent dye. Unless pumped out, the dye is trapped inside the cell, resulting in fluorescence intensity that is inversely proportional to P-glycoprotein activity. Briefly, cells harvested by trypsination were pre-incubated for 5 min in PBS containing 2% FBS. After Efluxx-ID Green dye was added, cells were incubated for 30 min and 10,000 cells underwent FACS analysis on a Calibur flow-cytometer using CellQuest software (Becton-Dickinson, NJ) using 490 nm excitation and 514 nm emission wavelengths.

### Measurement of cellular MMP and PMP

For MMP measurements, cells were seeded at densities of 5 x 10^4^ per well in a 96-well black plate with a transparent bottom. Cells were pre-incubated for 20 min at 37°C in culture media with FCCP or verapamil. Culture wells were replenished with 100 μl phenol red free RPMI containing 2% FBS and 500 nM of Mitotracker Red FM, a fluorescent dye that stains mitochondria of live cells as a function of mΔΨ. Cells were incubated for 30 min at 37°C in 5% CO_2_ and then washed with 100 μl of cold PBS per well. Fluorescence remaining in each well was measured on a microplate reader using 594 nm excitation and 642 nm emission wavelengths.

For PMP measurements, cells were seeded at densities of 5 x 10^4^ per well in a 96-well black plate with a transparent bottom. Culture wells were replenished with 100 μl PBS containing 5 μM of DiBAC4(3), a fluorescent dye that indicates plasma membrane potential of live cells. Cells were incubated for 30 min at 37°C in 5% CO_2_. Fluorescence in each well was measured on a microplate reader using 490 nm excitation and 510–580 nm emission wavelengths.

### Statistical analysis

All data are presented as mean ± SD of samples. Significance in difference between groups was analyzed by Student's t tests for 2 groups and ANOVA with Tukey post hoc tests for 3 or more groups. Relationships between ^99m^Tc-MIBI and MMP levels were assessed by linear correlation analyses with Pearson correlation coefficient measurements. *P* values <0.05 were considered statistically significant.

## Results

### MDR1 and MRP1 expression

**Fig 1:**
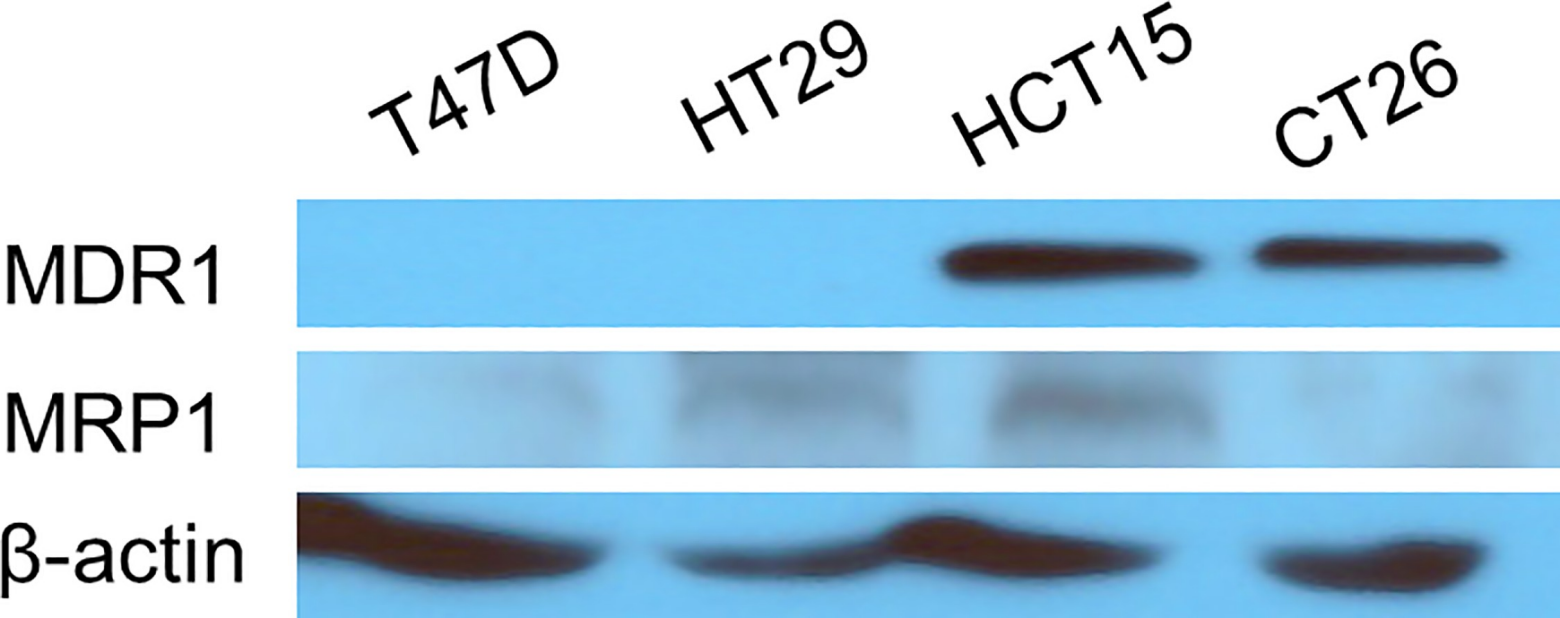
Western blots of MDR1 and MRP1 protein in 4 different cancer cell lines.

Western blots demonstrated low MDR1 expression in T47D breast cancer cells or HT29 colon cancer cells but high levels of MDR1 expression in HCT15 and CT26 colon cancer cells (Fig 1). In comparison, multidrug resistance-associated protein 1 (MRP1) expression was low in all of the 4 cancer cell lines, although small amounts of the protein were detected in HT29 and HCT15 cells (Fig 1).

### Effects of FCCP and verapamil on MMP of cancer cells with low MDR1 expression

**Fig 2:**
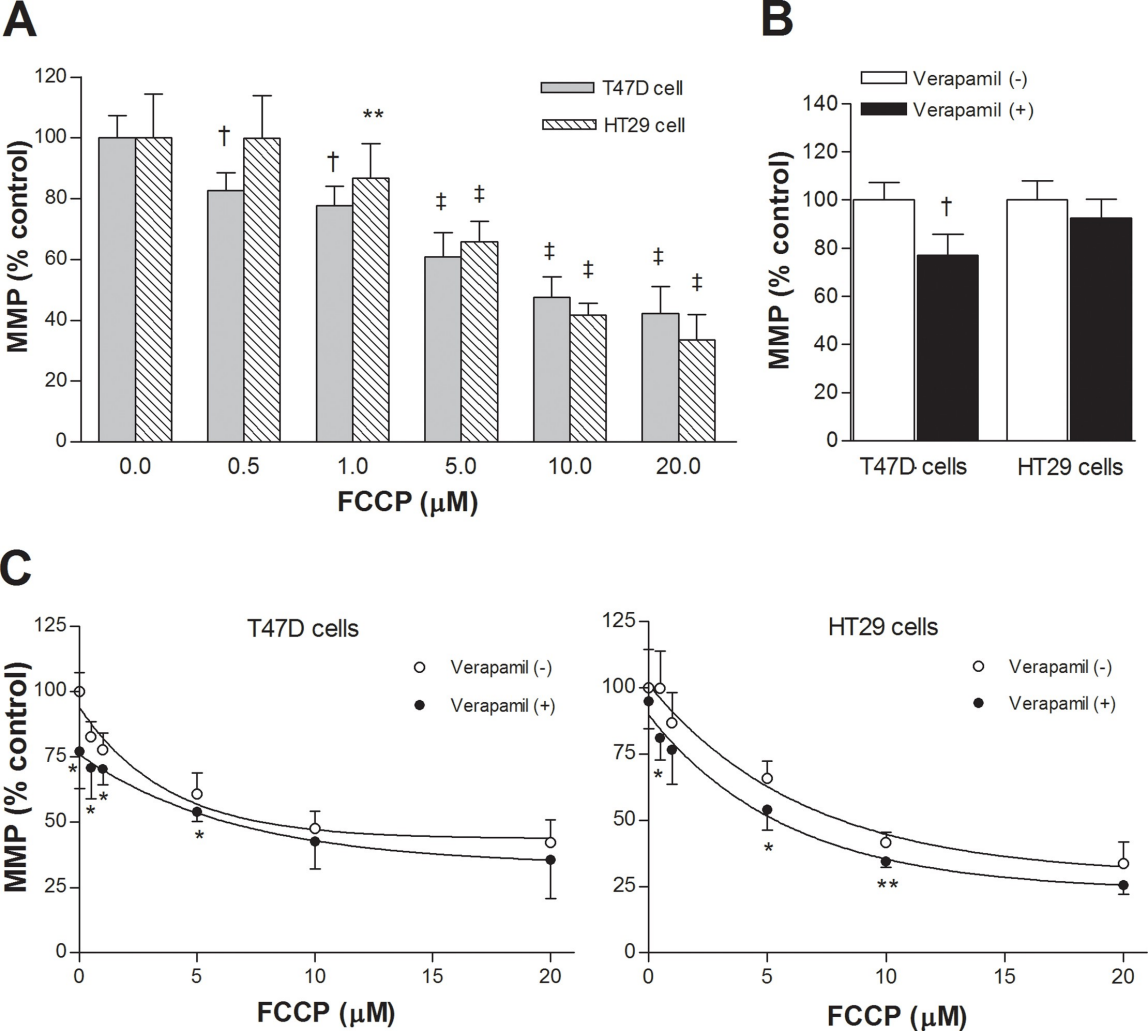
Effects of FCCP and verapamil on MMP of T47D and HT29 cancer cells. (A) Dose-dependent reduction of MMP by 20 min treatment with graded doses of FCCP. (B) Effect of 20 min treatment with 50 μM verapamil on MMP measurements. (C) MMP measurements in cancer cells treated with graded doses of FCCP with or without verapamil. Data are mean ± SD of 5 (A, C) or 4 (B) samples per group expressed as % of control level. ^‡^*P* <0.001; †*P* <0.005; **P <0.01; *P <0.05, compared to controls (A, B) or to cells treated with the same FCCP doses without verapamil (C).

T47D and HT29 cells displayed dose-dependent reductions of MMP by graded doses of FCCP (Fig 2A), reaching 42.3 ± 8.7% and 33.6 ± 8.3% of controls, respectively, by 20 μM. Residual MitotrackerRed activity in CT26 cells treated with 20 μM FCCP were shown to be localized in the cytosol by confocal microscopy (S1 Fig).

When we tested whether verapamil had any influence on MMP measurements, small reductions (77.1 ± 8.8%) or no significant influence (92.5 ± 7.9%) was found in respective cell types (Fig 2B). Minor influences of verapamil on MMP measurements for these cells were also observed during treatment with graded doses of FCCP (Fig 2C).

Repeated MMP measurements in HT29 cells using tetramethylrhodamine as an indicator instead of Mitotracker Red showed highly similar results. Hence, both indicators demonstrated that MMP was substantially reduced by 20 μM FCCP and mildly reduced by 20 μM verapamil.

### Effects of FCCP and verapamil on MMP in cancer cells with high MDR1 expression

**Fig 3:**
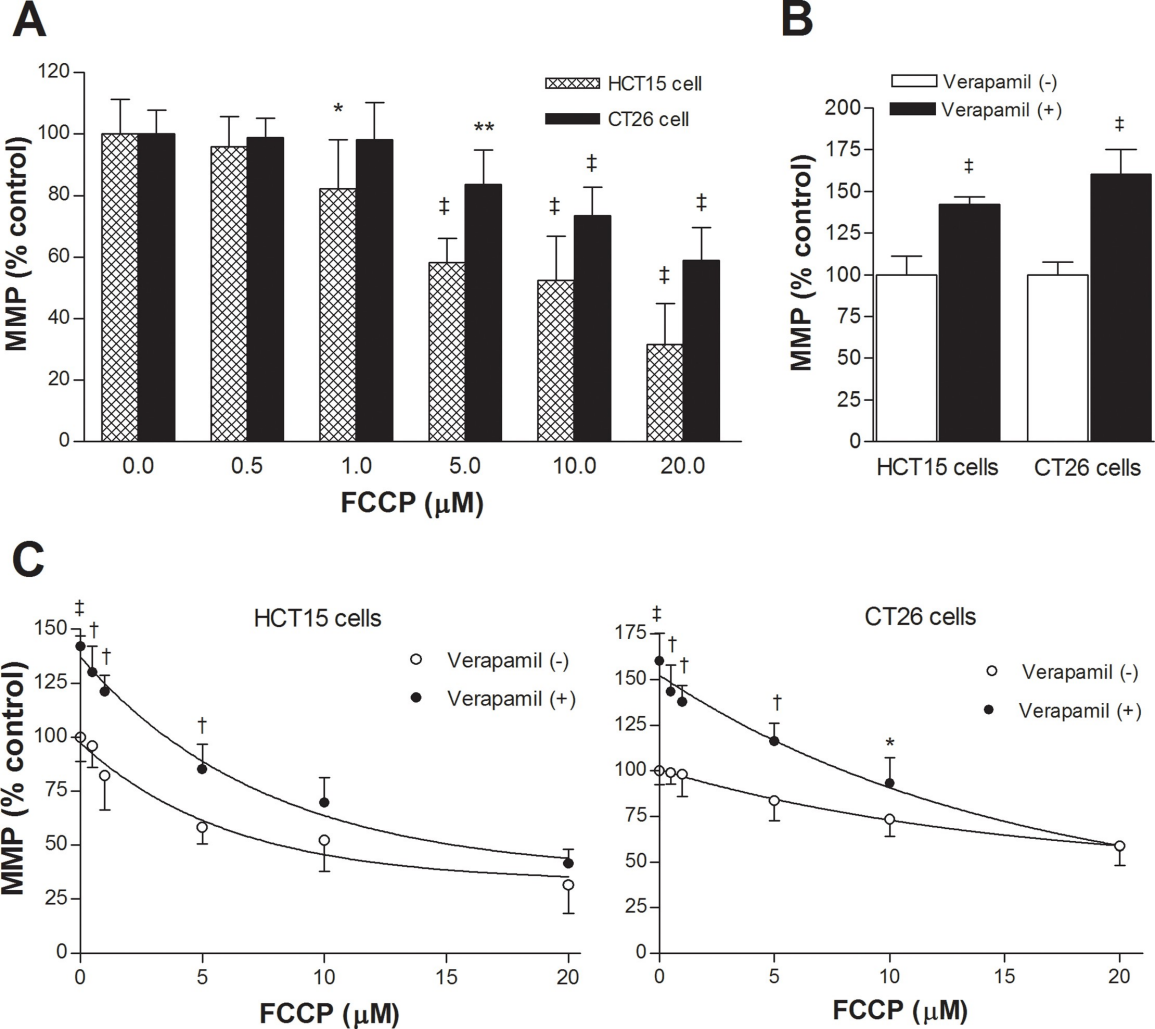
Effects of FCCP and verapamil on MMP of HCT15 and CT26 cancer cells. (A) Dose-dependent reduction of MMP by 20 min treatment with graded doses of FCCP. (B) Effect of 20 min treatment with 50 μM verapamil on MMP measurements. (C) MMP measurements in cancer cells treated with graded doses of FCCP with or without verapamil. Data are mean ± SD of 5 (A, C) or 4 (B) samples per group expressed as % of control level. ^‡^*P* <0.001; †*P* <0.005; **P <0.01; *P <0.05 compared to controls (A, B) or to cells treated with the same FCCP doses without verapamil (C).

HCT15 and CT26 cells that had high MDR1 expression also displayed dose-dependent reductions of MMP by graded doses of FCCP (Fig 3A). Thus, MMP was decreased to 31.7 ± 13.2% and 58.9 ± 6.4% of the respective controls by 20 μM of FCCP.

Verapamil significantly increased MMP measurements in HCT15 cells to 142.1 ± 4.7% and CT26 cells to 160.2 ± 15.0% of controls (both *P* <0.001; Fig 3B), indicating a small amount of Mitotracker Red FM efflux that was blocked by verapamil. Mild to modest elevations of MMP measurements by verapamil were also observed during treatment with graded FCCP doses. However, the differences became smaller under higher FCCP concentrations (Fig 3C).

### Effects of FCCP and verapamil on ^99m^Tc-MIBI uptake in cancer cells with low MDR1 expression

**Fig 4:**
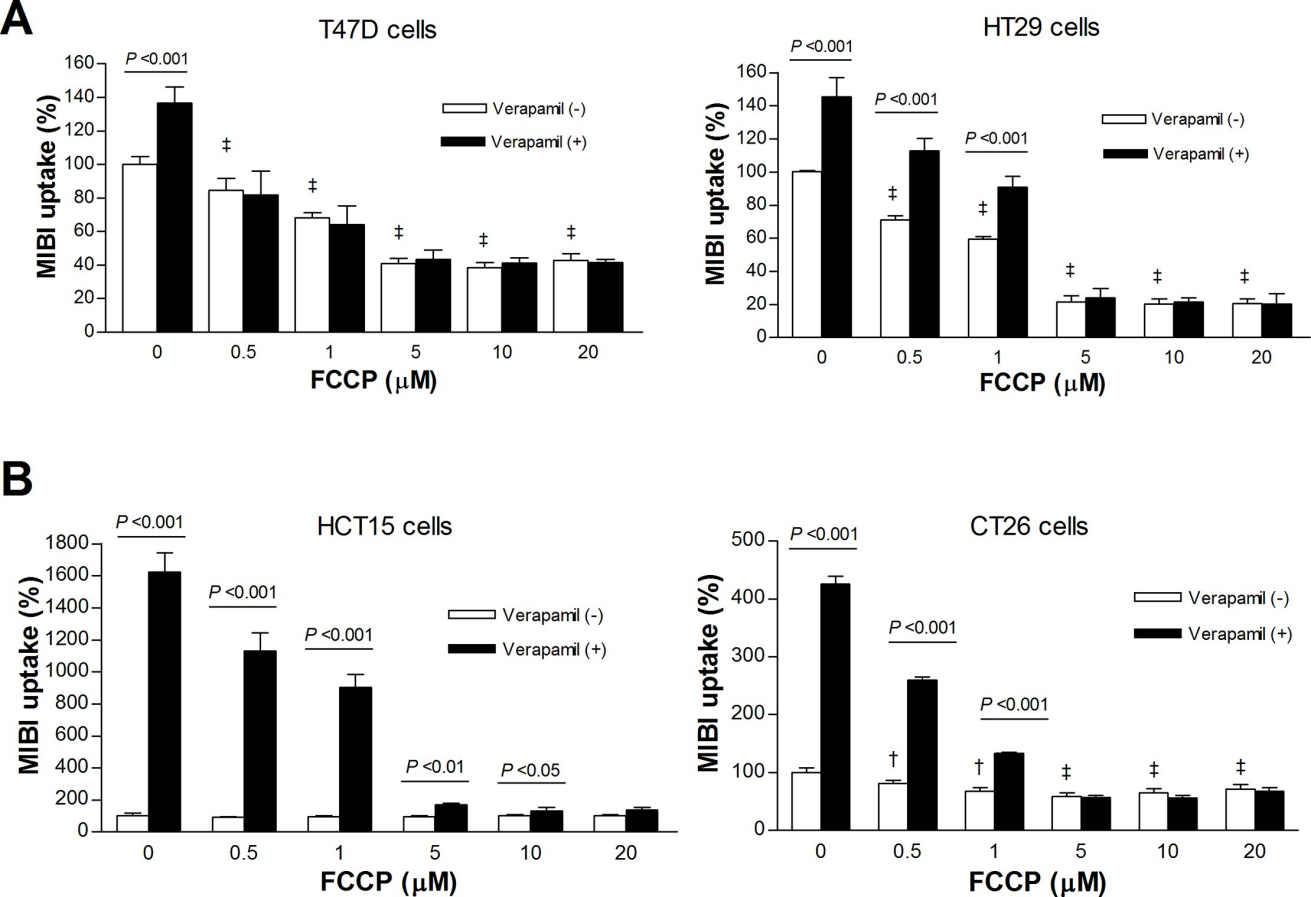
Effects of FCCP and verapamil on ^99m^Tc-MIBI uptake of cancer cells. Effects of graded doses of FCCP with or without verapamil on ^99m^Tc-MIBI accumulation for T47D and HT29 cells **(A)**, and for CT26 and HCT15 cells **(B)**. Bars are mean ± SD of 5 samples per group expressed as % of untreated cells. ^‡^*P* <0.001; †*P* <0.005, compared to untreated controls. Only *P* values for the verapamil (-) group are shown (left).

We next evaluated the effect of FCCP on cancer cell ^99m^Tc-MIBI accumulation. In T47D and HT29 cancer cells, FCCP caused significant dose-dependent reductions of ^99m^Tc-MIBI uptake (Fig 4A). At the FCCP concentration of 5 μM, ^99m^Tc-MIBI accumulation reached a lower plateau, reaching 40.8 ± 3.0% and 20.6 ± 5.6% of controls for respective cell types.

Verapamil treatment in the absence of FCCP mildly increased ^99m^Tc-MIBI uptake to 136.5 ± 9.8% and 145.3 ± 11.7% of controls, respectively (both *P* <0.001; Fig 4A). However, this effect became smaller as MMP was lowered by FCCP treatment and was completely lost with FCCP doses ≥5 μM (Fig 4A). Efflux of ^99m^Tc-MIBI through MRP was small as assessed in HT29 cells treated with 50 μM of MK-571 (S2 Fig).

**Fig 5:**
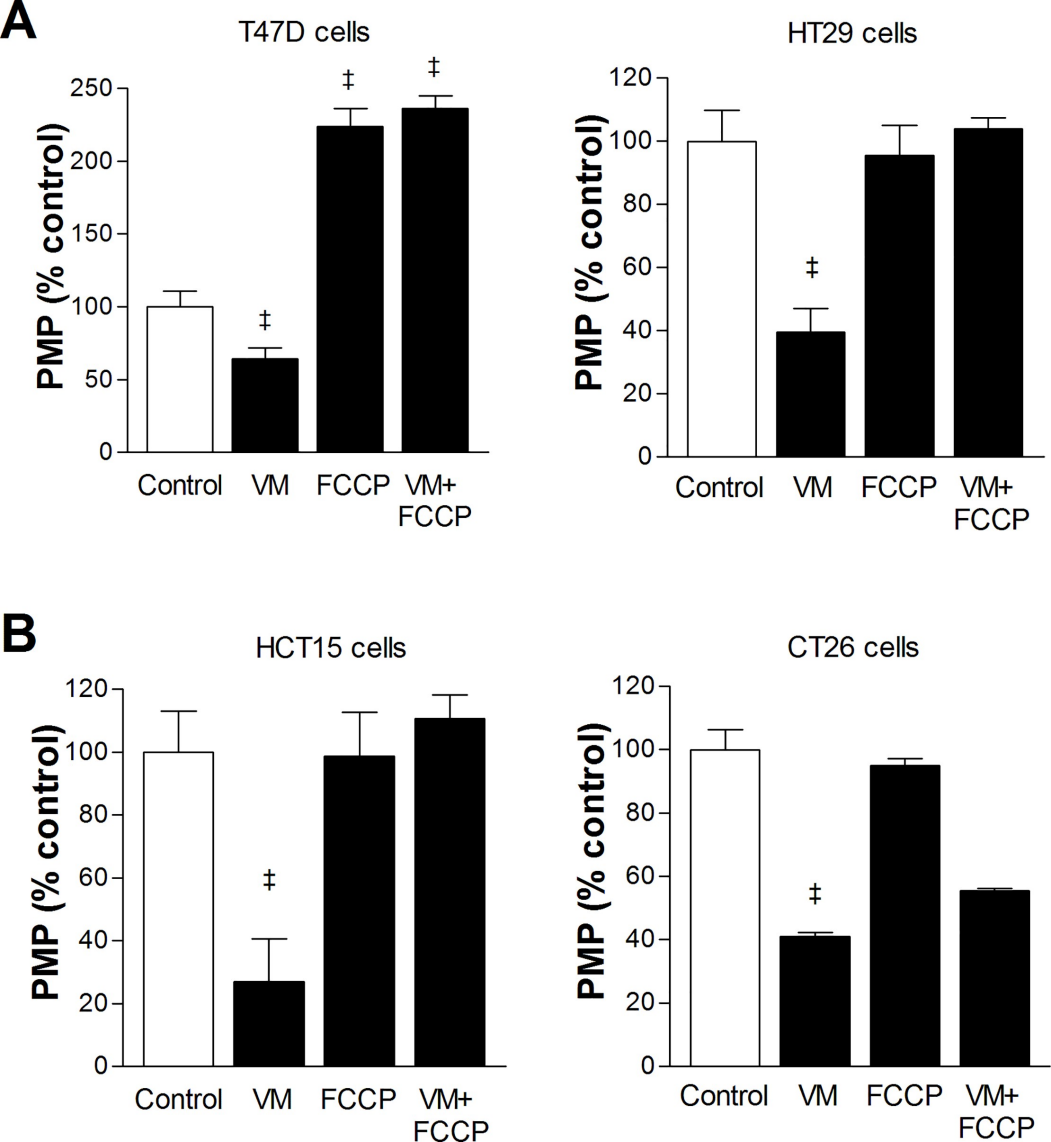
Effects of FCCP and verapamil on PMP of cancer cells. Effects of graded doses of FCCP with or without verapamil (VM) on PMP for T47D and HT29 cells **(A)**, and for CT26 and HCT15 cells **(B)**. Bars are mean ± SD of 5 samples per group expressed as % of untreated cells. ^‡^*P* <0.001; †*P* <0.005, compared to untreated controls.

When plasma membrane potential (PMP) was measured, verapamil was shown to significantly reduce PMP levels of T47D cells to 64.2 ± 7.7% and HT29 cells to 39.5 ± 7.6% of controls (both *P* <0.001; Fig 5A). FCCP increased PMP of T47D cells but did not influence PMP of HT29 cells. Fluorescence microscopy of HT29 cells confirmed that PMP was reduced by verapamil but was uninfluenced by FCCP (S3 Fig). In both cell types, FCCP completely abrogated the ability of verapamil to reduce PMP (Fig 5A). Together, the mild verapamil-stimulated ^99m^Tc-MIBI uptake and its reversal by FCCP in these cells might be explained by verapamil’s ability to reduce cellular PMP in a manner that is recovered by FCCP.

### Effects of FCCP and verapamil on ^99m^Tc-MIBI uptake in cancer cells with high MDR1 expression

In HCT15 and CT26 cancer cells, FCCP only slightly decreased ^99m^Tc-MIBI accumulation, if at all (Fig 4B). This was considered to represent greater radiotracer efflux through P-glycoprotein when MMP-mediated early uptake was higher.

Verapamil caused marked increases of ^99m^Tc-MIBI uptake in HCT15 cells to 4.2 ± 0.4-fold and in CT26 cells to 16.1 ± 1.7-fold of controls (both *P* < 0.001; Fig 4B). Again, this effect became smaller as MMP was lowered by FCCP treatment and was completely lost with FCCP doses ≥5 μM (Fig 4B). Efflux of ^99m^Tc-MIBI through MRP was small as assessed in CT26 cells treated with 50 μM of MK-571 (S2 Fig).

When Efluxx-ID Green multidrug resistance assay dye was used as a P-glycoprotein substrate independent of MMP, retention in CT26 cells was substantially increased by verapamil, consistent with suppression of P-glycoprotein activity. When MMP was reduced by graded doses of FCCP, cellular retention of this dye was not decreased as with ^99m^Tc-MIBI but was rather slightly increased (S4 Fig).

Verapamil treatment significantly reduced PMP of HCT15 cells to 40.9 ± 1.3% and of CT26 cells to 27.0 ± 19.5% of controls (both *P* < 0.001; Fig 5B). FCCP treatment did not influence PMP in either cell type. Fluorescence microscopy of CT26 cells confirmed that PMP was reduced by verapamil but was uninfluenced by FCCP (S4 Fig). Verapamil-suppressed PMP level was completely reversed by FCCP in CT26 cells, and partially reversed by FCCP in HCT15 cells (Fig 5B). Taken together, the ability of verapamil to reduce PMP might explain the excessive enhancement of ^99m^Tc-MIBI uptake by the drug in these cells.

To verify that our measurements were not affected by any changes in cell content after treatment, we first showed that Bradford assay-based protein content and SRB assay-based viable cell content of the four cancer cell lines were completely uninfluenced by treatment with FCCP and/or verapamil (S5 Fig). We further confirmed in a separate set of the four cancer cell lines that ^99m^Tc-MIBI uptake and MMP measurements normalized for protein content and viable cell content were highly similar to measurements obtained without such normalization (S6 Fig).

### Correlation between ^99m^Tc-MIBI accumulation and MMP in the four cancer cell lines

**Fig 6:**
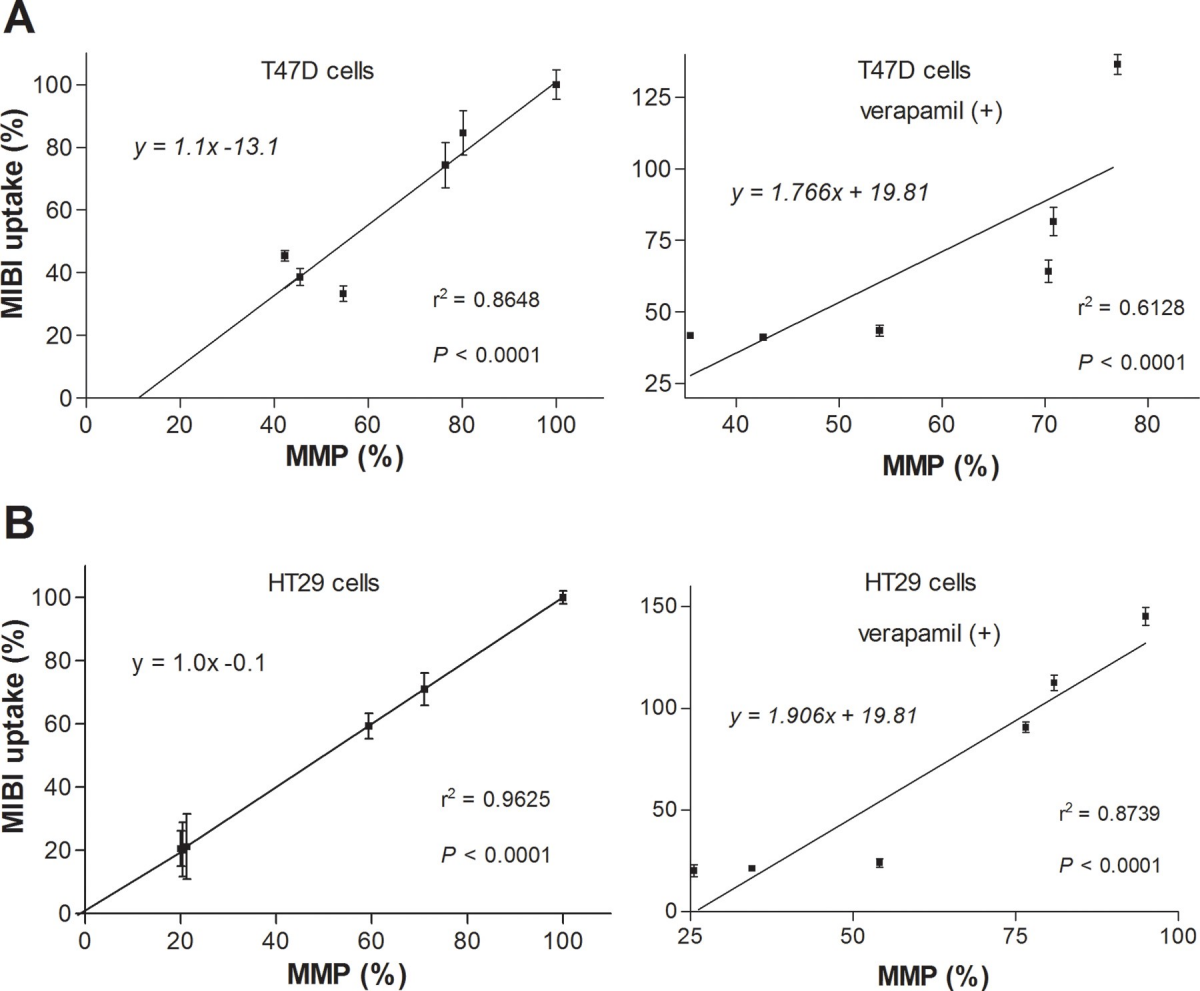
Relationship between ^99m^Tc-MIBI and MMP level in MDR1-negative cells. Linear regression curves for cellular ^99m^Tc-MIBI accumulation versus MMP level in T47D cells (A) and HT29 cells (B). r, Pearson’s correlation coefficient.

Finally, we compared the magnitude of ^99m^Tc-MIBI accumulation and MMP level. The results in T47D and HT29 cells that have low MDR1 expression revealed nearly perfect linear relationships with or without verapamil. Thus, correlation curves showed slopes near unity, near-zero *y* intercepts, and high r^2^ values of 0.8648 and 0.9625 without verapamil and 0.6128 and 0.8739 with verapamil, respectively (both *P* <0.0001; Fig 6).

**Fig 7:**
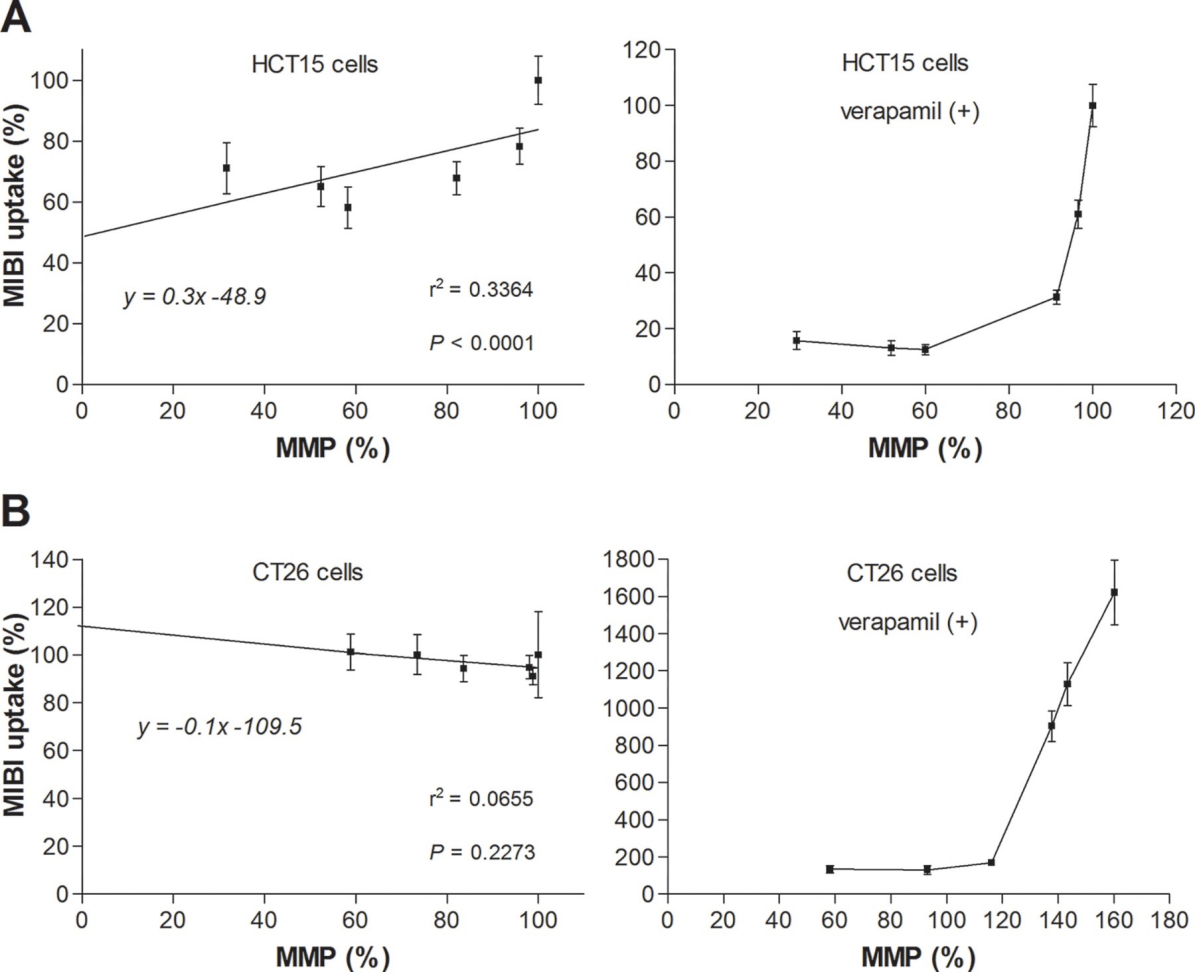
Relationship between ^99m^Tc-MIBI and MMP level in MDR1-positive cells. (**A, B**) Linear regression curves for cellular ^99m^Tc-MIBI accumulation versus MMP level in HCT15 cells (**A**) and CT26 cells (**B**) in the absence of verapamil (left) and relation curves in the presence of verapamil (right). r, Pearson’s correlation coefficient.

However, linear correlation curves of HCT15 and CT26 cells that have high MDR1 expression displayed shallow slopes with high *y* intercepts (Fig 7A and 7B), which likely represented greater ^99m^Tc-MIBI efflux as early uptake increased through higher MMP. In these cells, when efflux was blocked with verapamil, ^99m^Tc-MIBI accumulation increased markedly as MMP level rose. However, the rate of increased ^99m^Tc-MIBI accumulation became excessive when the MMP rose above a certain level (Fig 7A and 7B).

### Correlation between ^99m^Tc-MIBI accumulation and MMP level in three PDCs

**Fig 8:**
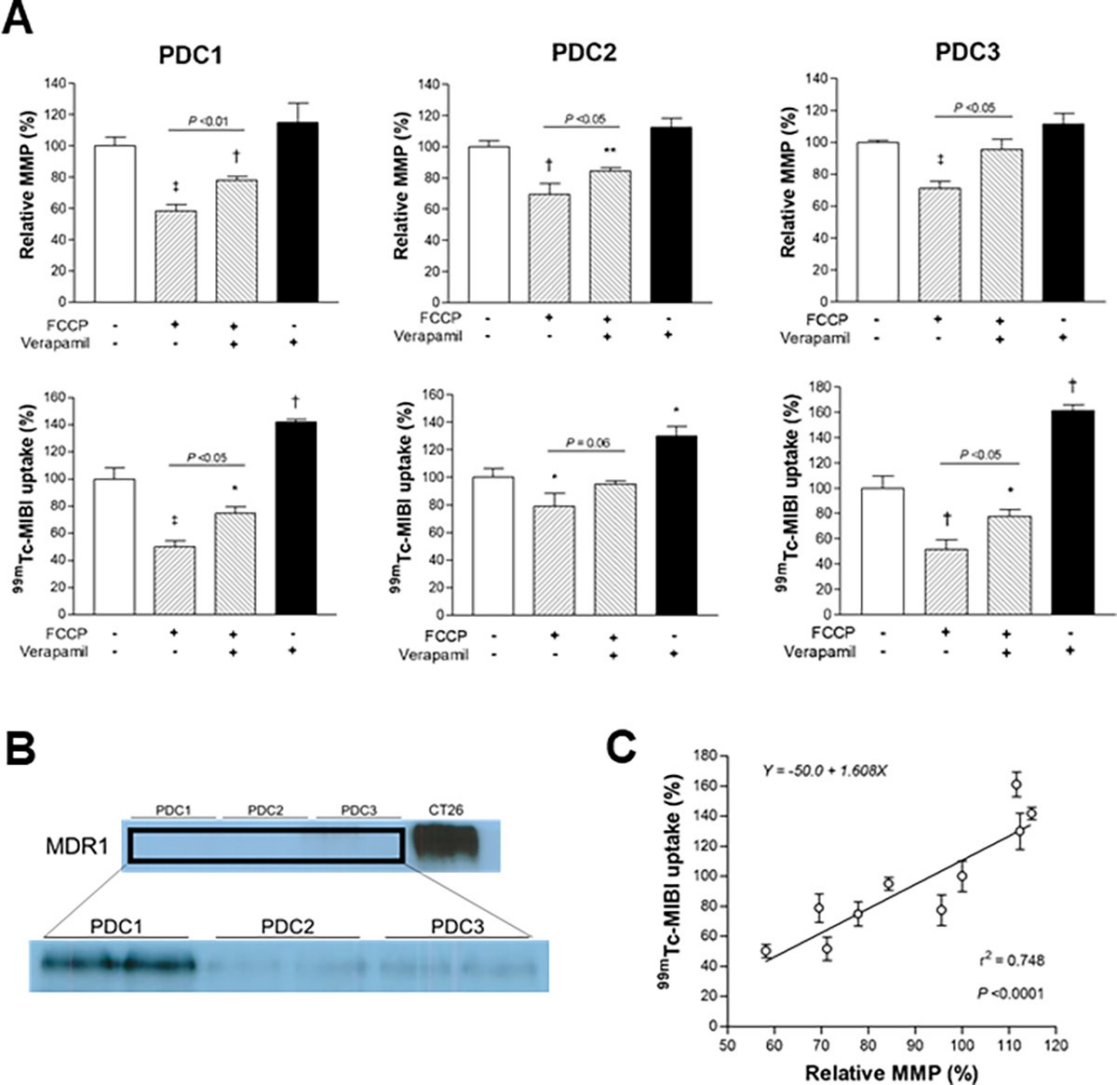
Effects of FCCP and verapamil on MMP and ^99m^Tc-MIBI accumulation in PDCs. (A) MMP (top) and ^99m^Tc-MIBI uptake (bottom) in three different colon cancer PDCs at baseline and after treatment with FCCP and/or verapamil. (B) Western blots of MDR1 protein in the PDCs compared to high-expressing CT26 cells, and again in the PDCs with greater protein loading. (C) Linear correlation between relative MMP and ^99m^Tc-MIBI uptake levels. Data are mean ± SD of protein assay-corrected MMP and SRB assay-corrected ^99m^Tc-MIBI uptake from triplicate samples expressed as % of untreated controls. *P <0.05; **P <0.01; †P <0.005; ‡P <0.001, compared to controls.

Additional evaluation of three different colon cancer PDCs verified the findings obtained from the cancer cell lines. Thus, FCCP significantly suppressed MMP of the three PDCs to 58.2 ± 4.2%, 69.5 ± 7.1% and 71.2 ± 4.5% of controls, respectively. FCCP similarly reduced ^99m^Tc-MIBI accumulation to 50.3 ± 4.4%, 78.8 ± 9.5% and 51.7 ± 7.7% of controls, respectively (Fig 8A). Verapamil partially recovered both MMP and ^99m^Tc-MIBI accumulation that was lowered by FCCP, and it slightly increased ^99m^Tc-MIBI accumulation when used alone (Fig 8A). Western blots showed negligible MDR1 band intensities in all three PDCs compared to the prominent MDR1 band intensity of high expressing CT26 cells. However, repeated blots with greater protein loading showed that the PDCs actually had low to modest but significant amounts of MDR1 expression (Fig 8B). When we compared ^99m^Tc-MIBI accumulation to relative MMP in these cells, a high linear correlation with an r value of 0.865 (r square, 0.748) was found (Fig 8C).

## Discussion

Although the use of ^99m^Tc-MIBI in oncology has largely been focused on its role as a substrate for P-glycoprotein-mediated efflux, the optimized use of this tracer for tumor imaging could benefit from a better understanding of how tumor accumulation is influenced by MMP. Given the key role of MMP in tumor metabolism and survival, and the need of malignant cells need to maintain their MMP elevated at a certain range [13–15], this might provide opportunities to evaluate tumor MMP with ^99m^Tc-MIBI under certain circumstances.

MMP level of cultured cells are readily measured by quantifying fluorescent intensity from accumulation of permeant lipophilic dyes that bind to mitochondria via the membrane potential. Using this technique, we first showed that FCCP dose-dependently reduced the MMP of cancer cells. On our MMP assays, cells treated with 20 μM of FCCP showed about 40% of MitotrackerRed retention compared to untreated cells, but this was shown to represent specific MMP signals rather than nonspecific cell binding since confocal imaging localized the residual dye within the cytosol. We next sought to assess the ability of ^99m^Tc-MIBI to monitor MMP changes during treatment with graded doses of FCCP by measuring its cellular accumulation. However, since cells with high MDR1 levels need to be tested while P-glycoprotein-mediated ^99m^Tc-MIBI efflux is blocked, we first assessed the influence of verapamil on cellular MMP. The results demonstrated that verapamil exerted little influence on MMP in cells with low MDR1 expression, although it significantly increased MMP measurements in cells with high MDR1 expression. We interpreted this to indicate that Mitotracker Red FM is a weak substrate for P-glycoprotein-mediated efflux. Such an effect was recently demonstrated for the cationic MMP dye JC-1 [26]. When we added graded doses of FCCP, the magnitude of increased MMP measurements by verapamil became smaller. Hence, measurement of higher MMP using this dye was mildly influenced by P-glycoprotein-mediated efflux.

We next investigated the effects of graded FCCP doses on ^99m^Tc-MIBI accumulation in the presence or absence of verapamil. In cells with low MDR1 expression, FCCP dose-dependently reduced ^99m^Tc-MIBI accumulation that closely simulated the patterns of MMP reduction. When FCCP dose reached 5 μM, ^99m^Tc-MIBI level in these cells reached a low plateau, which represents MMP-independent accumulation. A previous study showed that carbonyl cyanide m-chlorophenyl hydrazone (CCCP) could lead to the release of over 70% of ^99m^Tc-MIBI taken up by tumor cells [27]. Our results also indicate that 20–40% of baseline ^99m^Tc-MIBI accumulation was MMP-independent. In contrast, cells with high MDR1 expression displayed minimal reduction in ^99m^Tc-MIBI accumulation by graded FCCP doses. This suggests that higher early ^99m^Tc-MIBI uptake underwent greater P-glycoprotein-mediated efflux. In CT26 cells, graded doses of FCCP did not decrease, but rather slightly increased retention of Efluxx-ID Green assay dye, a P-glycoprotein substrate independent of MMP.

When we repeated uptake experiments in the presence of verapamil, cells with high MDR1 expression displayed marked increases of ^99m^Tc-MIBI accumulation as FCCP concentrations decreased. Yet, the magnitude of increased ^99m^Tc-MIBI accumulation was disproportionately high compared to that of MMP. In addition, the presence of verapamil also modestly increased ^99m^Tc-MIBI accumulation in cells with low MDR1 expression. A possible mechanism for these findings could be the drug’s strong hydrophobicity that can influence cytoplasmic membrane fluidity [28, 29]. An increase of bilayer fluidity by verapamil has been suggested to facilitate passive diffusion of substrates into cells [30]. Our results of reduced PMP by verapamil in a manner that was recovered by FCCP supports this possibility. However, differences in PMP responses according to cell types may represent cellular differences in biologic properties other than MDR1 expression. ^99m^Tc-MIBI enters the cytoplasm by passing through the cell membrane via passive transport. Therefore, verapamil might increase cancer cell ^99m^Tc-MIBI accumulation by both facilitating its passive transport through the cell membrane and blocking P-glycoprotein-mediated efflux.

Our data demonstrated a near perfect linear relationship between ^99m^Tc-MIBI and MMP level in cells with low MDR1 expression. Cells with high MDR1 expression displayed a flattened ^99m^Tc-MIBI response to MMP changes in the absence of verapamil. In the presence of verapamil, these cells showed a disproportionally marked increase of ^99m^Tc-MIBI accumulation as MMP level rose. Although both ^99m^Tc-MIBI and Mitotracker Red are sensitive to MMP and P-glycoprotein-mediated efflux, a key difference is that MIBI is used in tracer concentration whereas MitotrackerRed is more highly concentrated, which may have contributed to the different responses of the two probes.

Additional evaluation of three different colon cancer PDCs demonstrated that ^99m^Tc-MIBI accumulation is highly dependent on MMP level unless MDR1 expression is extremely high as in CT26 or HCT15 cell lines. Indeed, these PDCs had low to modest levels of MDR1 protein, but linear correlation analysis revealed a high r square indicating that 74.8% of ^99m^Tc-MIBI accumulation in the cells might be explained by differences in MMP.

## Conclusions

Low baseline ^99m^Tc-MIBI uptake that is markedly increased by verapamil represents cancer cells with high levels of MDR1 expression. However, in cancer cells with low or modest levels of MDR1 expression that do not markedly increase ^99m^Tc-MIBI uptake by verapamil, the magnitude of uptake is largely dependent on cellular MMP. This finding may be helpful when interpreting ^99m^Tc-MIBI tumor imaging. Future investigations in murine models are thus warranted to clarify the significance of this finding in tumors of living bodies.

## Supporting information

S1 Fig Confocal fluorescence microscopic imaging of MMP using MitotrackerRed on CT26 cancer cells. Confocal fluorescence image of MDR1-positive CT26 cells with 20 μM FCCP. MDR1 inhibitors (verapamil) and FCCP after the PMP assay. Red color is MitotrackerRed, blue is DAPI. Magnification, x1000.
(DOCX)

S2 Fig Effects of ABC transporter inhibitors on MIBI uptake in CT26 and HT29 cancer cells. MIBI uptake of MDR1-positive CT26 cells and MDR1-negative HT29 cells in the presence of the MDR1 inhibitor verapamil (Vera; *20 μM*), MRP inhibitor MK571 (MK; 50 μM), and BCRP inhibitor novobiocin (Novo; 200 μM), compared to untreated control cells. Data are mean ± SD of 3 samples per group expressed as % of control level.
(DOCX)

S3 Fig Fluorescence microscopic imaging of PMP in CT26 and HT29 cancer cells. Fluorescent images of MDR1-positive CT26 and MDR1-negative HT29 cells with or without MDR1 inhibitors (verapamil) or FCCP after incubation with the PMP assay dye. Magnification, x40.
(DOCX)

S4 Fig Measurement of P-glycoprotein activity. P-glycoprotein activity was measured by FACS analysis using Efluxx-ID Green multidrug resistance assay dye as a substrate independent of MMP. In CT26 cells, dye retention was substantially increased by 20 μM verapamil, consistent with suppression of p-glycoprotein activity. Cellular retention of this dye was not decreased when MMP was reduced by graded doses of FCCP.
(DOCX)

S5 Fig Protein and cell contents are uninfluenced by FCCP and/or verapamil treatment. (A,B) Effects of FCCP and/or verapamil on Bradford assay-based protein content (A) and SRB assay-measured viable cell content (B) in various colon cancer cells. Bars are mean ± SD of 5 samples per group expressed as % of untreated controls.
(DOCX)

S6 Fig Effects of FCCP and verapamil on protein and cell content-corrected 99mTc-MIBI uptake and MMP. (A,B) Effects of FCCP and/or verapamil on SRB assay-corrected MMP (A) and Bradford assay-corrected 99mTc-MIBI accumulation (B) in various colon cancer cells. Bars are mean ± SD of 5 samples per group expressed as % of untreated controls. *P <0.05; **P <0.01; †P <0.005; ‡P <0.001, compared to controls.
(DOCX)
