# Supplementary material for: 99mTc-MIBI uptake as a marker of mitochondrial membrane potential in cancer cells and effects of MDR1 and verapamil
Source: PLoS One. 2020 Feb 12;15(2):e0228848. doi: 10.1371/journal.pone.0228848 (PMC7015412; doi:10.1371/journal.pone.0228848)
Supplement: S1 Fig — Confocal fluorescence image of MDR1-positive CT26 cells with 20 μM FCCP. MDR1 inhibitors (verapamil) and FCCP after the PMP assay. Red color is MitotrackerRed, blue is DAPI. Magnification, x1000. (DOCX) [file pone.0228848.s001.docx]

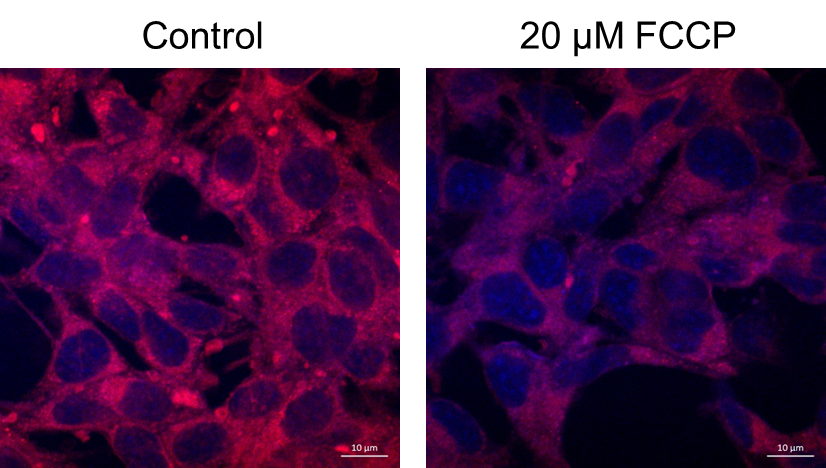


**Supplementary Fig. 1. Confocal fluorescence microscopic imaging of MMP using MitotrackerRed on CT26 cancer cells.** Confocal fluorescence image of MDR1-positive CT26 cells with 20 μM FCCP. MDR1 inhibitors (verapamil) and FCCP after the PMP assay. Red color is MitotrackerRed, blue is DAPI. Magnification, x1000.
