# Supplementary material for: 99mTc-MIBI uptake as a marker of mitochondrial membrane potential in cancer cells and effects of MDR1 and verapamil
Source: PLoS One. 2020 Feb 12;15(2):e0228848. doi: 10.1371/journal.pone.0228848 (PMC7015412; doi:10.1371/journal.pone.0228848)
Supplement: S2 Fig — MIBI uptake of MDR1-positive CT26 cells and MDR1-negative HT29 cells in the presence of the MDR1 inhibitor verapamil (Vera; 20 μM), MRP inhibitor MK571 (MK; 50 μM), and BCRP inhibitor novobiocin (Novo; 200 μM), compared to untreated control cells. Data are mean ± SD of 3 samples per group expressed as % of control level. (DOCX) [file pone.0228848.s002.docx]

**Supplementary Fig. 2. Effects of ABC transporter inhibitors on MIBI uptake in CT26 and HT29 cancer cells.** MIBI uptake of MDR1-positive CT26 cells and MDR1-negative HT29 cells in the presence of the MDR1 inhibitor verapamil (Vera; *20 μM*), MRP inhibitor MK571 (MK; 50 μM), and BCRP inhibitor novobiocin (Novo; 200 μM), compared to untreated control cells. Data are mean ± SD of 3 samples per group expressed as % of control level.
