# Supplementary material for: 99mTc-MIBI uptake as a marker of mitochondrial membrane potential in cancer cells and effects of MDR1 and verapamil
Source: PLoS One. 2020 Feb 12;15(2):e0228848. doi: 10.1371/journal.pone.0228848 (PMC7015412; doi:10.1371/journal.pone.0228848)
Supplement: S3 Fig — Fluorescent images of MDR1-positive CT26 and MDR1-negative HT29 cells with or without MDR1 inhibitors (verapamil) or FCCP after incubation with the PMP assay dye. Magnification, x40. (DOCX) [file pone.0228848.s003.docx]

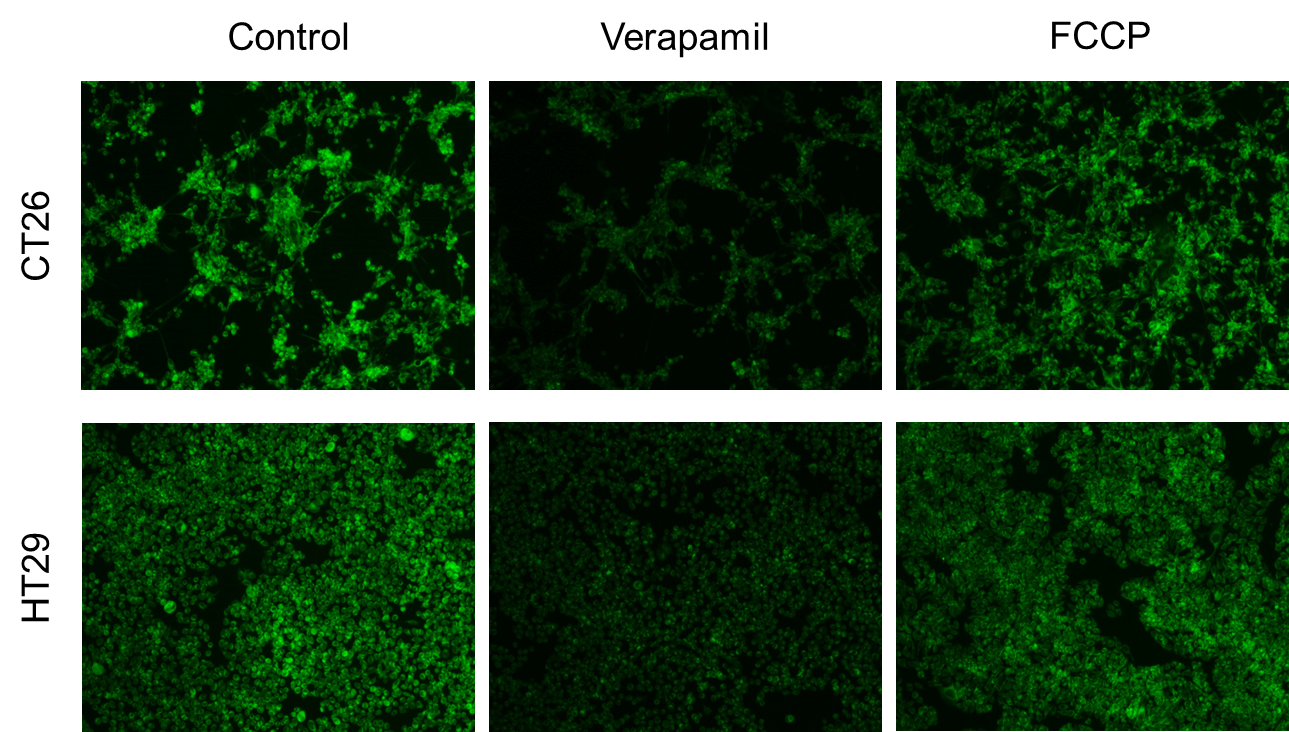


**Supplementary Fig. 3. Fluorescence microscopic imaging of PMP in CT26 and HT29 cancer cells.** Fluorescent images of MDR1-positive CT26 and MDR1-negative HT29 cells with or without MDR1 inhibitors (verapamil) or FCCP after incubation with the PMP assay dye. Magnification, x40.
