# Supplementary material for: 99mTc-MIBI uptake as a marker of mitochondrial membrane potential in cancer cells and effects of MDR1 and verapamil
Source: PLoS One. 2020 Feb 12;15(2):e0228848. doi: 10.1371/journal.pone.0228848 (PMC7015412; doi:10.1371/journal.pone.0228848)
Supplement: S4 Fig — P-glycoprotein activity was measured by FACS analysis using Efluxx-ID Green multidrug resistance assay dye as a substrate independent of MMP. In CT26 cells, dye retention was substantially increased by 20 μM verapamil, consistent with suppression of p-glycoprotein activity. Cellular retention of this dye was not decreased when MMP was reduced by graded doses of FCCP. (DOCX) [file pone.0228848.s004.docx]

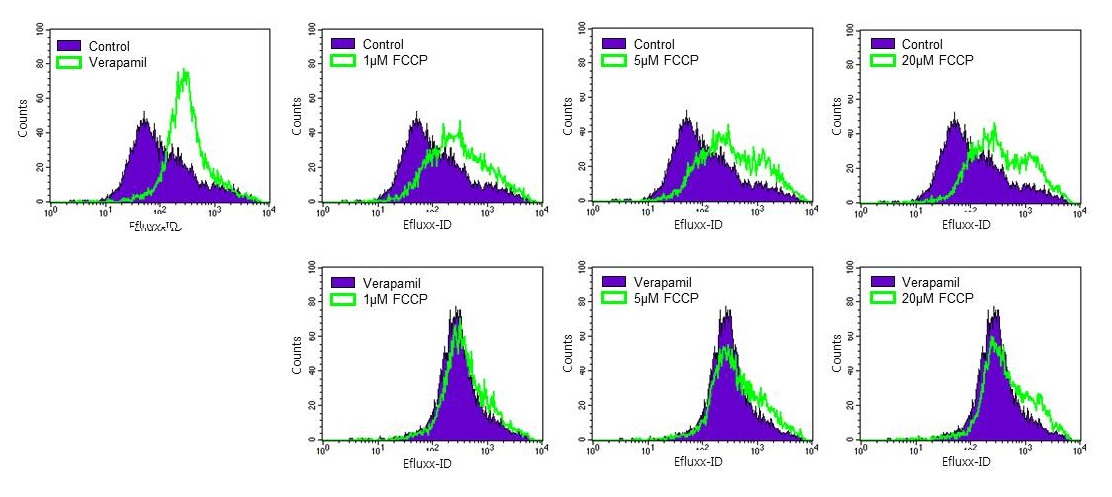


**Supplementary Fig. 4. Measurement of P-glycoprotein Activity.** P-glycoprotein activity was measured by FACS analysis using Efluxx-ID Green multidrug resistance assay dye as a substrate independent of MMP. In CT26 cells, dye retention was substantially increased by 20 μM verapamil, consistent with suppression of p-glycoprotein activity. Cellular retention of this dye was not decreased when MMP was reduced by graded doses of FCCP.
