# Supplementary material for: 99mTc-MIBI uptake as a marker of mitochondrial membrane potential in cancer cells and effects of MDR1 and verapamil
Source: PLoS One. 2020 Feb 12;15(2):e0228848. doi: 10.1371/journal.pone.0228848 (PMC7015412; doi:10.1371/journal.pone.0228848)
Supplement: S5 Fig — (A,B) Effects of FCCP and/or verapamil on Bradford assay-based protein content (A) and SRB assay-measured viable cell content (B) in various colon cancer cells. Bars are mean ± SD of 5 samples per group expressed as % of untreated controls. (DOCX) [file pone.0228848.s005.docx]

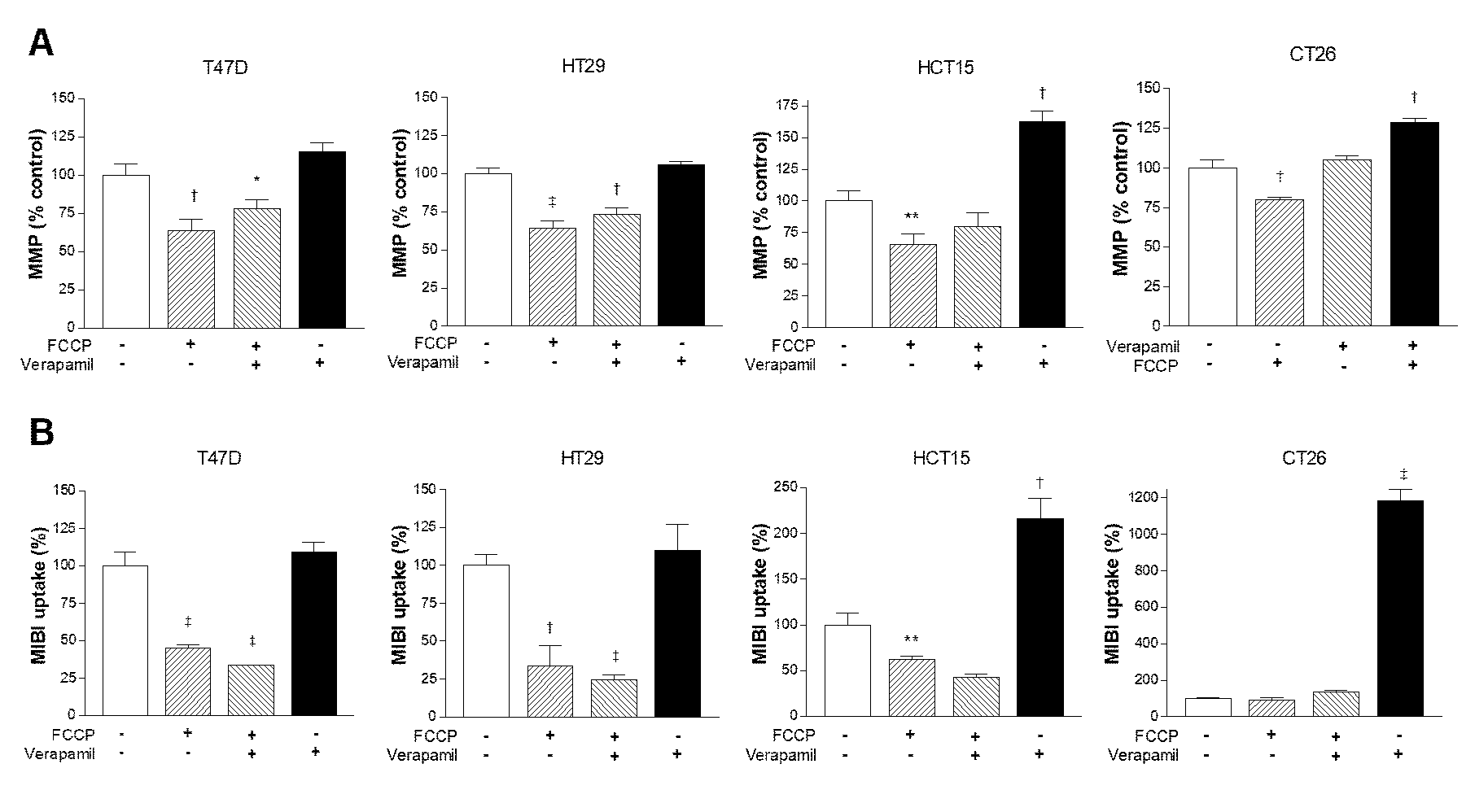


**Supplementary Fig. 5**. **Protein and cell contents are uninfluenced by FCCP and/or verapamil treatment.** (A,B) Effects of FCCP and/or verapamil on Bradford assay-based protein content (A) and SRB assay-measured viable cell content (B) in various colon cancer cells. Bars are mean ± SD of 5 samples per group expressed as % of untreated controls.
