# Supplementary material for: 99mTc-MIBI uptake as a marker of mitochondrial membrane potential in cancer cells and effects of MDR1 and verapamil
Source: PLoS One. 2020 Feb 12;15(2):e0228848. doi: 10.1371/journal.pone.0228848 (PMC7015412; doi:10.1371/journal.pone.0228848)
Supplement: S6 Fig — (A,B) Effects of FCCP and/or verapamil on SRB assay-corrected MMP (A) and Bradford assay-corrected 99mTc-MIBI accumulation (B) in various colon cancer cells. Bars are mean ± SD of 5 samples per group expressed as % of untreated controls. *P <0.05; **P <0.01; †P <0.005; ‡P <0.001, compared to controls. (DOCX) [file pone.0228848.s006.docx]

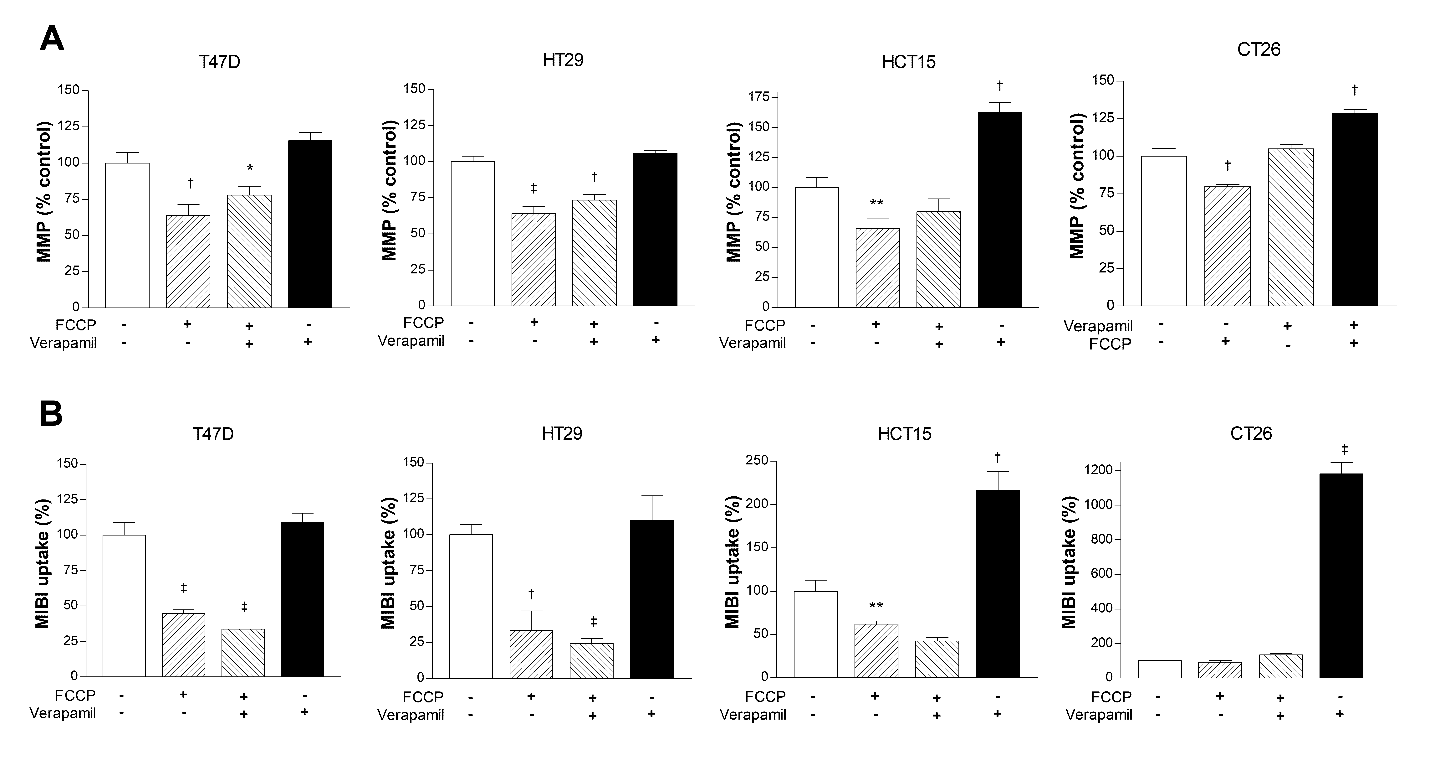


**Supplementary Fig. 6. Effects of FCCP and verapamil on protein and cell content-corrected 99mTc-MIBI uptake and MMP.** (A,B) Effects of FCCP and/or verapamil on SRB assay-corrected MMP (A) and Bradford assay-corrected 99mTc-MIBI accumulation (B) in various colon cancer cells. Bars are mean ± SD of 5 samples per group expressed as % of untreated controls. *P <0.05; **P <0.01; †P <0.005; ‡P <0.001, compared to controls.
